# Allicin inhibits the biological activities of cervical cancer cells by suppressing circEIF4G2

**DOI:** 10.1002/fsn3.3935

**Published:** 2024-01-04

**Authors:** Mao Yifan, Xu Rui, Li Yuan, Jiang Feiyun

**Affiliations:** ^1^ Gynecology of the Second People's Hospital of Wuhu City Wuhu China; ^2^ Department of Geriatrics The First Affiliated Hospital of Wannan Medical College Wuhu China; ^3^ Department of Gynecology Wuhu City Second People's Hospital Wuhu China

**Keywords:** AKT, allicin, circEIF4G2, Hela, HOXA1, mTOR, Siha

## Abstract

Allicin is a safe herbal extract believed to have antitumor effects, which, however, remain unclear. The aim of the present work was to discuss Allicin antitumor effects on cervical cancer using cell experiments. Using Hela and Siha to our research objectives in our study, first step, difference concentration of Allicin (20, 40, and 80 μM) treated Hela and Siha cell lines, and next step, discuss circEIF4G2 effects in Allicin antitumor effects in Hela and Siha cell lines; the cell proliferation and EdU‐positive cell number by CCK‐8 and EdU staining; cell apoptosis rate by flow cytometry; invasion cell number by transwell assay; wound healing rate by wound healing assay; and relative mRNA and protein levels using qRT‐PCR and WB assay. With Allicin supplement, the cell proliferation and EdU‐positive cell number were significantly depressed with cell apoptosis rate significantly increasing; invasion cell number and wound healing rate significantly suppressed with circEIF4G2 mRNA expression significantly down‐regulation (*p* < .05, respectively). However, there was no significant difference among Allicin, si‐circEIF4G2, and Allicin+si‐circEIF4G2 in cell biological activities including cell proliferation, apoptosis, invasion and migration, and relative gene and protein expression. Allicin depresses biological activities of cervical cancer cells through down‐regulating circEIF4G2/HOXA1/AKT/mTOR.

## Graphical abstract

Allicin inhibits biological activities of cervical cancer cells through down‐regulating circEIF4G2/HOXA1/AKT/mTOR.

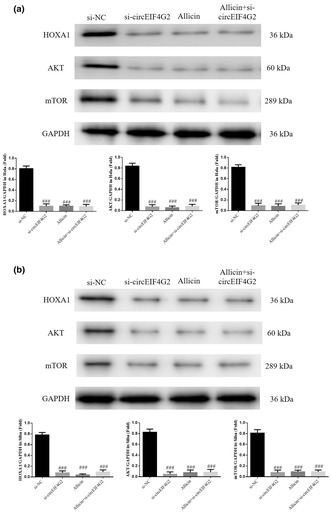

## 1 INTRODUCTION

Cervical cancer (CC) has a high incidence and mortality. CC accounts for 80% of the total incidence of tumors in female reproductive organs, and approximately 604,000 new CC cases are recorded worldwide annually, of which 342,000 die from this malignancy (Igissinov et al., 2021). Early CC can be treated with surgical resection; for advanced CC, the standard treatment is mainly radiotherapy and chemotherapy, but their long‐term use can easily lead to drug resistance (Shaswati et al., 2023; Tomizawa et al., 2020). Therefore, efficient anti‐CC drugs must be explored. Allicin is an active compound extracted from garlic. Fresh garlic contains no allicin, but when it is processed and crushed, alliinase is activated, catalyzing and decomposing alliin into allicin. The medicinal value of allicin has been relatively recognized, but its inhibitory effect on tumor cell proliferation remains unconfirmed (Alamir & Patil, 2021; Maitisha et al., 2021). Its regulatory mechanism on tumors is also unclear. CircRNAs are a class of single‐stranded, circular, closed, noncoding RNA molecules characterized by unsuitable degradation by ribonuclease, sequence conservation, stable expression, and tissue specificity (Chen et al., 2021; Su et al., 2019; Zhang & Dai, 2022). An increasing number of studies have shown that circRNAs have a potential application value in markers for cancer diagnosis and therapeutic targets (Chen et al., 2019; Yu & Liu, 2019). As a member of circRNAs, circEIF4G2 effectively inhibited biological activities of CC cells after knockdown in our previous study (Mao et al., 2019). In the present study, we wanted to explore whether allicin depresses the biological activities of CC cells (Hela and Siha, which are human papillomavirus‐positive cell lines) and whether it exerts its effect by down‐regulating circEIF4G2 expression.

## 2 MATERIALS AND METHODS

Main reagents: Allicin (95% purity) from Nanjing Zelang Medical Technology Co., Ltd. We prepared different allicin working solutions at 20, 40, and 80 μM concentrations by using the DMEM/F‐12 medium. Furthermore, cell apoptosis, cell counting kit‐8, and two‐step reverse transcription polymerase chain reaction assay kits purchased from Nanjing KeyGEN BioTech Co., Ltd., Beyotime Institute of Biotechnology in Jiangsu and Beijing Tiangen BioTech Co., Ltd., respectively. Primers and internal reference (glyceraldehyde 3‐phosphate dehydrogenase [GAPDH]) were synthesized from Sangon Biotech Co., Ltd., Shanghai. HOXA1, AKT, and primary anti‐mTOR antibodies were all purchased from Abcam, and si‐negative control (NC) and si‐circEIF4G2 were designed and synthesized from Nanjing KeyGEN BioTech Co., Ltd.

### 2.1 Cell culture

HeLa and Siha cells were preserved in our laboratory. The culture system was DMEM/F‐12, which contained 10% fetal bovine serum. The cells were cultured at 37°C and 5% CO_2_, with medium replacement once a day and cell passage once every 2–3 days. Cells at the logarithmic growth phase were then collected for subsequent experiments.

### 2.2 Cell transfection

HeLa and Siha (2 × 10^5^) cells were transfected with si‐NC or si‐circEIF4G2 by using Lipofectamine® 3000 according to grouping requirements depending on the manufacturer's protocol. After 48 h culture, the cells were collected for the next experiment.

### 2.3 Cell grouping

The cells were grouped into the following: NC (routinely treated group), dimethyl sulfoxide (DMSO) (culture medium supplemented with DMSO), and allicin‐L, allicin‐M, and allicin‐H (culture medium supplemented with allicin dissolved in DMSO at final concentrations of 20, 40, and 80 μM, respectively). We also divided the cells as follows: si‐NC (cells transfected with si‐NC using Lipofectamine® 3000), si‐circEIF4G2 (cells transfected with si‐circEIF4G2 using Lipofectamine® 3000), allicin (culture medium supplemented with allicin dissolved in DMSO at a final concentration of 80 μM), and allicin+si‐circEIF4G2 (cells transfected with si‐circEIF4G2 by Lipofectamine® 3000 and treated with allicin at a final concentration of 80 μM). After receiving the corresponding treatment for 48 h, the cells in each group were collected for subsequent experiments.

### 2.4 Cell proliferation by CCK‐8 assay

After undergoing the corresponding treatment for 48 h, the cells in each group were added with 10 μL of CCK‐8 stock solution. Following a continuous cell culture for 1 h, we measured the absorbance value (OD value) in each well by using a microplate reader and then calculated the cell proliferation inhibition rate. This experiment was repeated thrice.

### 2.5 Gene expression detection by quantitative RT‐PCR (qRT‐PPCR)

After 48 h of culture, cells were collected from the incubator and washed twice using phosphate‐buffered saline solution (PBS). After adding 1 mL of cell lysate TRIzol in each well, we extracted total RNA to determine RNA concentration and purity by using a microspectrophotometer. Then, cDNA was reverse‐transcribed and synthesized, followed by amplification. Table 1 shows the primer sequences. The reaction system consisted of the following: SYBR® Premix Ex Taq TM II, 5 μL; upstream primer, 0.25 μL; downstream primer, 0.25 μL; ROX, 0.2 μL; and cDNA, 2.5 μL (total volume: 10 μL). Amplification conditions were as follows: pre‐denaturation at 95°C for 30 s, denaturation at 95°C for 30 s, annealing at 57°C for 10 s, and extension at 72°C for 15 s (40 cycles). GAPDH was used as an internal reference, and the relative expression of the target gene was calculated by the 2^−ΔΔCt^ method.

**TABLE 1:** The primer sequences.

Gene name	F:(5′‐3′)	R:(5′‐3′)
circEIF4G2	TTTTTCAACAAAGCAAGGTCAA	TCTAGGTCCCACTGTCCTCA
HOXA1	GGGTGTCCTACTCCCACTCA	GGACCATGGGAGATGAGAGA
AKT	ATGCTGGACAAGGACGGG	CACGATGTTGGCAAAGAA
mTOR	GGCTTCTGAAGATGCTGTCC	GAGTTCGAAGGGCAAGAGTG
GAPDH	GGTGAAGGTCGGTGTGAACG	GCTCCTGGAAGATGGTGATGG

### 2.6 Flow cytometry

After the corresponding treatment for 48 h, cells were added into 0.25% pancreatin for digestion, centrifuged at 2000 r/min for 5 min, resuspended with 0.5 mL 2% PBS, and fixed with 1.5 mL of 100% ethanol on the ice at 4°C overnight. After centrifugation at 1000 r/min for 5 min, cells were washed with 3 mL of 2% PBS, followed by a water bath in 100 μL of RNase A at 37°C for 30 min and uniform staining with 400 μL of propidium iodide at 4°C in the dark for 30 min. Thereafter, we determined apoptosis by flow cytometry and recorded red fluorescence at 488 nm excitation wavelength. Data were analyzed using CellQuest.

### 2.7 EdU staining

Cells from each group were seeded into a 96‐well plate at 1 × 10^5^ cells/well and cultured overnight. Afterward, each well was added with 100 μL of culture medium containing 50 μmol/L EdU for incubation for 2 h and then washed twice with PBS for 5 min each time. After fixing and staining, we observed these cells under a fluorescence microscope.

### 2.8 Cell invasion capability by transwell assay

We applied a layer of Matrigel matrix above a semipermeable membrane, filled the lower chamber with culture medium containing trace supplements, inserted it with a transwell insert, and added treated cell suspension (1 × 10^5^ cells) to the upper chamber. Subsequently, the transwell system was incubated in a cell incubator for 48 h. After cell suspension removal, the upper chamber was washed using PBS. In the lower chamber, we removed the culture medium, added 800 μL of methanol, which was fixed at room temperature for 30 min, and then washed thrice using PBS. Afterward, this chamber was placed in a solution premixed with 0.1% crystal violet for reaction at room temperature for 30 min. After being washed three times using PBS, the cells were counted and photographed under an inverted microscope.

### 2.9 Wound healing assay

After the corresponding treatment for 48 h, the migration capability of cells in each group was assessed by wound healing assay. Cells in the logarithmic growth phase were collected from each group, scratched perpendicular to a cell culture plate using a pipette tip, and then washed thrice using PBS. At 0 and 48 h, we photographed and measured the width of the wound, respectively.

### 2.10 Western blotting (WB) assay

Relevant protein levels in the cells of each group were evaluated using the WB assay. After adding protease inhibitors, we quantified the proteins through a bicinchoninic acid assay. Subsequently, we added and boiled sample buffer for 5 min and separated the proteins by using SDS‐PAGE, transferred them to a PVDF membrane, and blocked them in 5% skim milk for 1 h. Afterward, primary antibodies were added for incubation overnight. After washing the membrane thrice, we added secondary antibodies, followed by incubation for 1 h, membrane washing three times, and finally, enhanced chemiluminescence development. The gray value was analyzed using Gel‐Pr32.

### 2.11 Statistical methods

Expressed as mean ± SD, data were analyzed and processed using SPSS 22.0. We used the *F* test and LSD‐*t* test for multigroup and pairwise comparisons of measurement data, respectively. A *p*‐value <.05 was considered statistically significant.

## 3 RESULTS

### 3.1 Allicin on Hela and Siha cell proliferation

**FIGURE 1:**
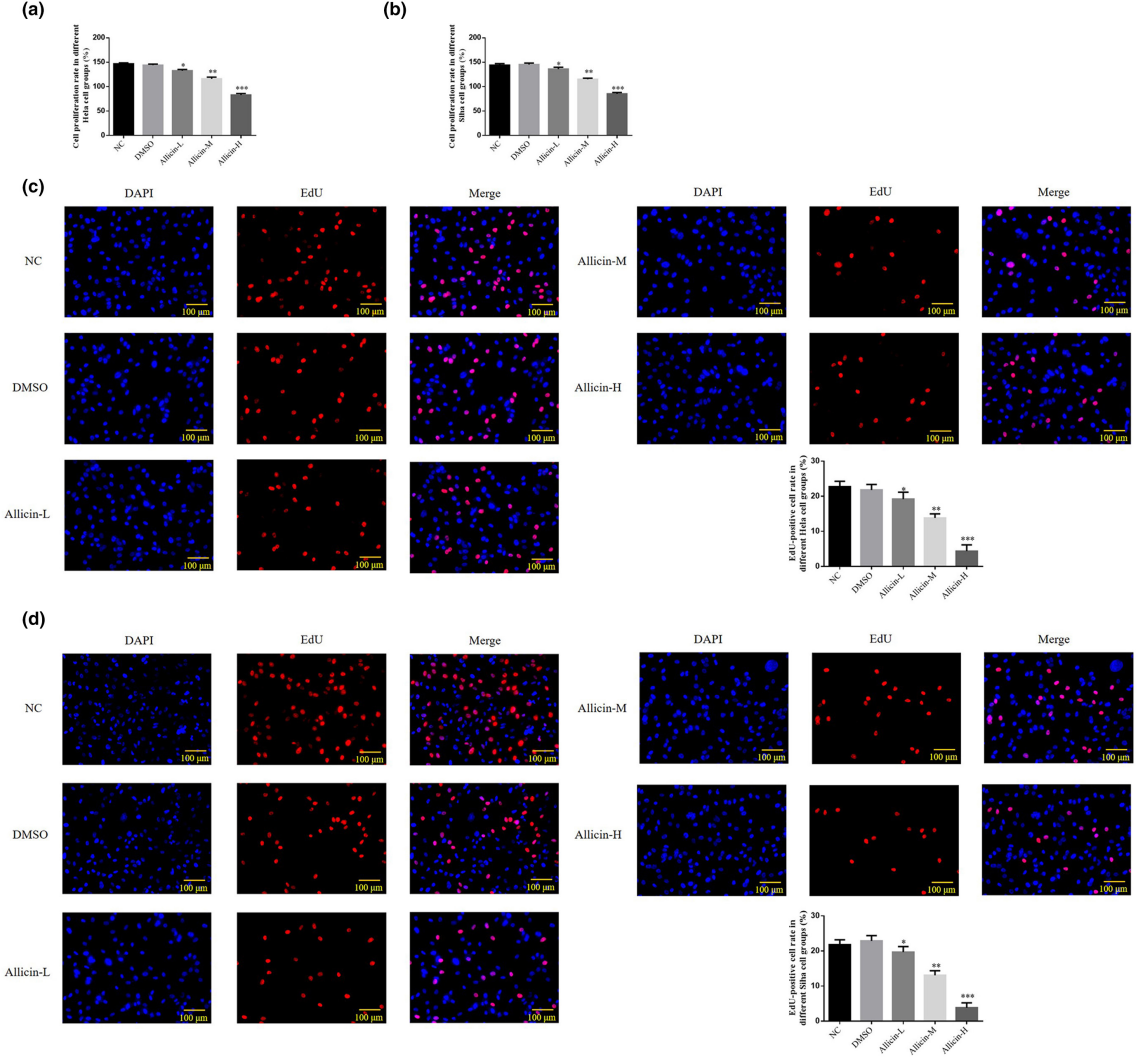
Effects of allicin on proliferation of Hela and Siha cells. (a) Cell proliferation in different Hela cell groups. (b) Cell proliferation in different Siha cell groups. (c) EdU‐positive Hela cell number (×100). (d) EdU‐positive Siha cell number (×100). Allicin‐H, The cells were treated with 80 μ M Allicin; Allicin‐L, The cells were treated with 20 μ M Allicin; Allicin‐M, The cells were treated with 40 μ M Allicin; DMSO, The cells were treated with DMSO; NC, The cells were treated with normal. *p < .05; **p < .01; ***p < .001, compared with NC group.

CCK‐8 assay and EdU staining showed no significant difference in cell proliferation rate or EdU‐positive cell count in the DMSO group (DMSO's concentration is 0.25%) (*p* > .05, Figure 1), indicating that DMSO is nontoxic to Hela and Siha cells. After allicin treatment, the cell proliferation rate (Figure 1a,b) and EdU‐positive cell count (Figure 1c,d) reduced significantly in the allicin‐L, allicin‐M, and allicin‐H groups (*p* < .05, *p* < .01, or *p* < .001, Figure 1a,d). Taken together, 0.25% DMSO concentration was safe for Hela and Siha cell lines, and allicin‐L, allicin‐M, and allicin‐H decreased Hela and Siha cell proliferation, with allicin‐H showing the best effect.

### 3.2 Allicin on Hela and Siha cell apoptosis

**FIGURE 2:**
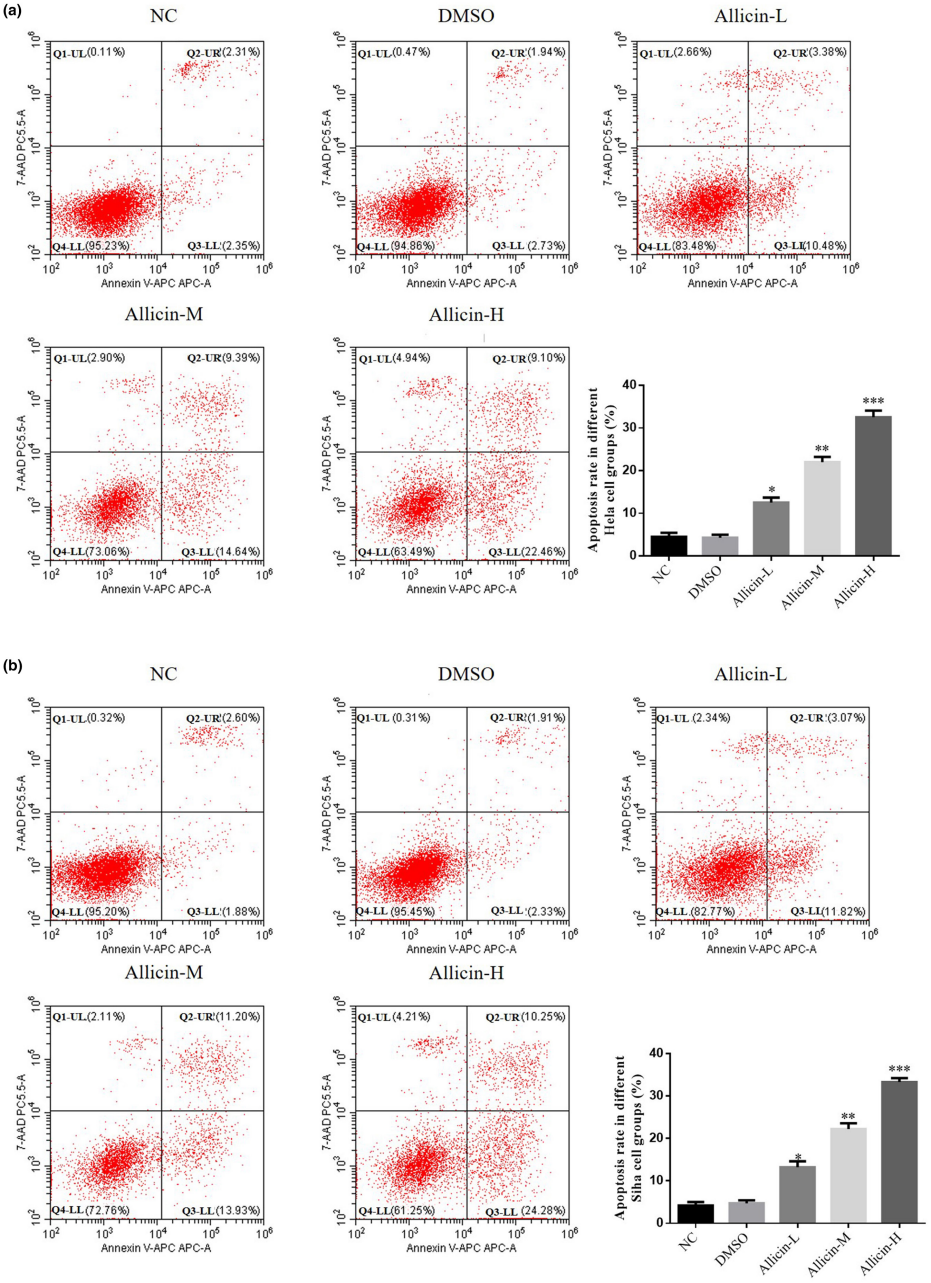
Effects of Allicin on apoptosis of Hela and Siha cells. Allicin‐H, The cells were treated with 80 μ M Allicin; Allicin‐L, The cells were treated with 20 μ M Allicin; Allicin‐M, The cells were treated with 40 μ M Allicin; DMSO, The cells were treated with DMSO; NC, The cells were treated with normal. (a) Apoptosis rate of different Hela cell groups. (b) Apoptosis rate of different Siha cell groups. *p < .05; **p < .01; ***p < .001, compared with NC group.

According to flow cytometry results, the apoptotic rate in DMSO group presented no significant difference (*p* > .05, Figure 2), suggesting that DMSO is nontoxic to Hela and Siha cells. After allicin treatment, apoptotic rate in the allicin‐L, allicin‐M, and allicin‐H groups increased significantly (*p* < .05, *p* < .01, or *p* < .001, Figure 2a,b). Overall, 0.25% DMSO concentration was safe for Hela and Siha cell lines, and allicin‐L, allicin‐M, and allicin‐H increased apoptosis of Hela and Siha cells, with allicin‐H exhibiting best effect.

### 3.3 Allicin on invasion capability of Hela and Siha cells

**FIGURE 3:**
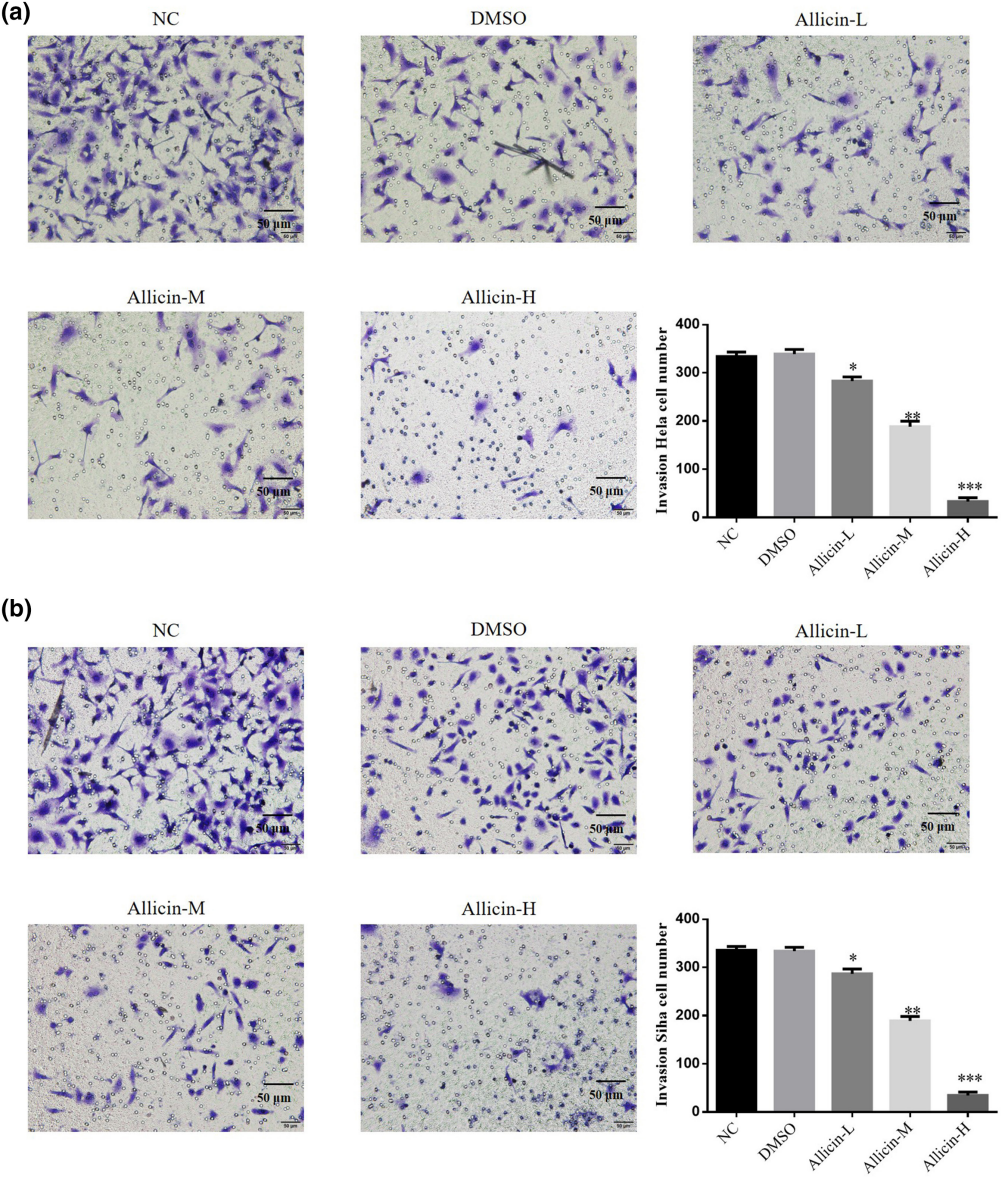
Effects of allicin on invasion capability of Hela and Siha cells. Allicin‐H, The cells were treated with 80 μ M Allicin; Allicin‐L, The cells were treated with 20 μ M Allicin; Allicin‐M, The cells were treated with 40 μ M Allicin; DMSO, The cells were treated with DMSO; NC, The cells were treated with normal. (a) Invasion Hela cell number by transwell assay (×100). (b) Invasion Siha cell number by transwell assay (×100). *p < .05; **p < .01; ***p < .001, compared with NC group.

Transwell assay revealed no significant difference in the number of invasive cells in the DMSO group (*p* > .05, Figure 3), indicating that DMSO has no effect on Hela and Siha cells' invasion capability. After allicin treatment, the invasive cell number in the allicin‐L, allicin‐M, and allicin‐H groups decreased significantly (*p* < .05, *p* < .01, or *p* < .001, Figure 3a,b). Taken together, allicin‐L, allicin‐M, and allicin‐H decreased Hela and Siha cells' invasion ability, with allicin‐H showing best effect.

### 3.4 Allicin on migration capability of Hela and Siha cells

**FIGURE 4:**
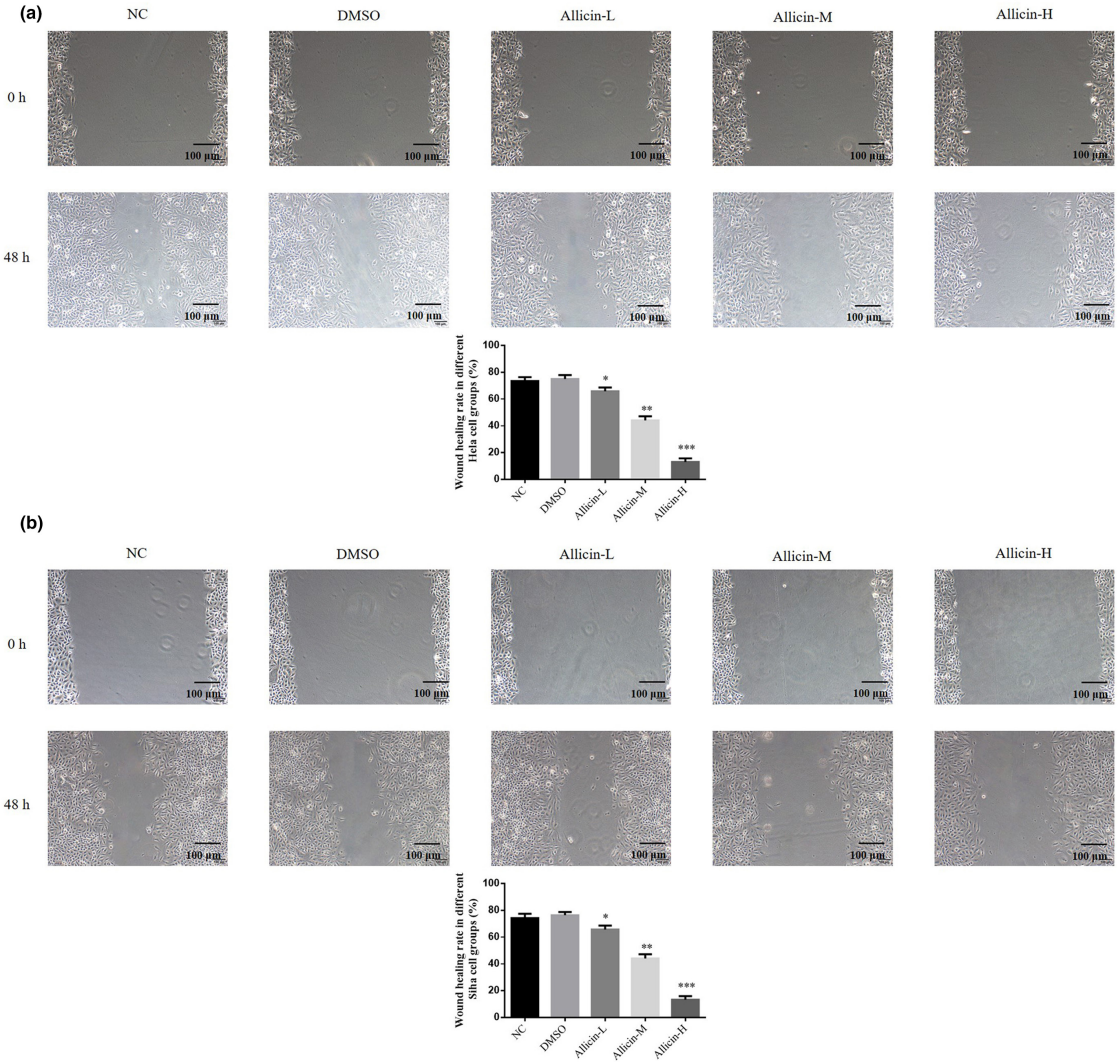
Effects of Allicin on migration capability of Hela and Siha cells. Allicin‐H, The cells were treated with 80 μ M Allicin; Allicin‐L, The cells were treated with 20 μ M Allicin; Allicin‐M, The cells were treated with 40 μ M Allicin; DMSO, The cells were treated with DMSO; NC, The cells were treated with normal. (a) Wound healing rate in different Hela cell groups (×100). (b) Wound healing rate in different Siha cell groups (×100). *p < .05; **p < .01; ***p < .001, compared with NC group.

Wound healing assay results revealed the wound healing rate did not significantly differ between the DMSO and NC groups (*p* > .05, Figure 4), suggesting DMSO has no effect on Hela and Siha cells' migration capability. After allicin treatment, wound healing rate in allicin‐L, allicin‐M, and allicin‐H groups decreased significantly (*p* < .05, *p* < .01, or *p* < .001, Figure 4a,b). Thus, allicin‐L, allicin‐M, and allicin‐H decreased Hela and Siha cells' migration ability, with best effect observed in allicin‐H.

### 3.5 Effect of allicin on circEIF4G2 gene expression

**FIGURE 5:**
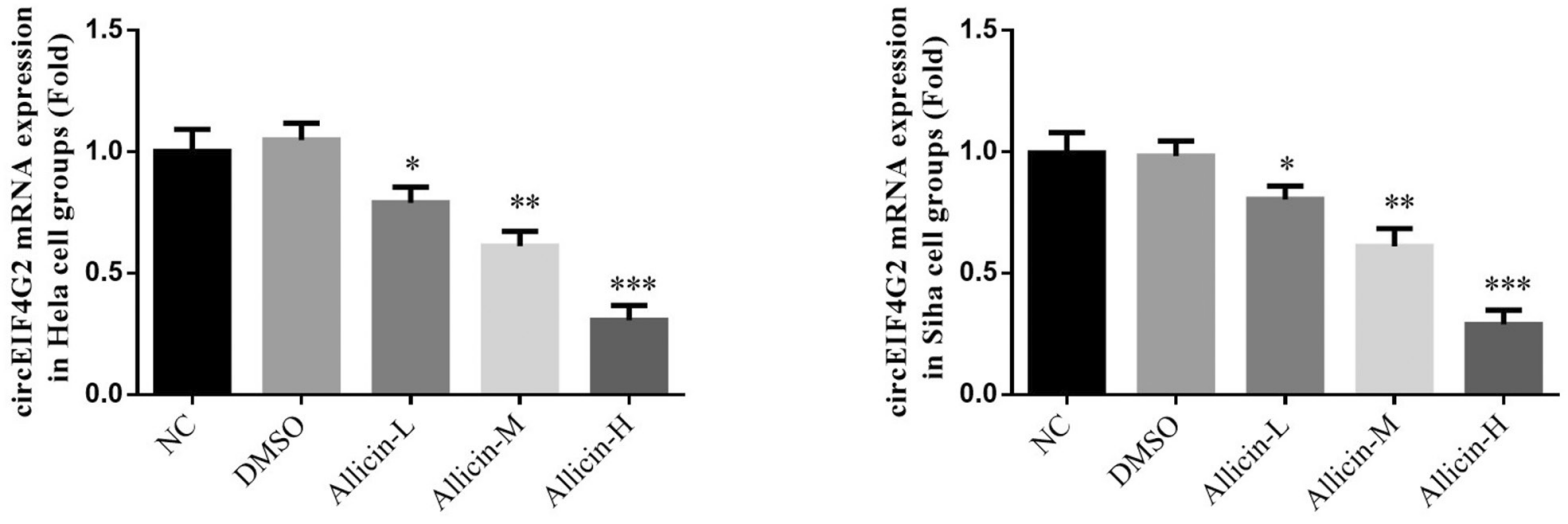
Effect of Allicin on circEIF4G2 gene expression. Allicin‐H, The cells were treated with 80 μ M Allicin; Allicin‐L, The cells were treated with 20 μ M Allicin; Allicin‐M, The cells were treated with 40 μ M Allicin; DMSO, The cells were treated with DMSO; NC, The cells were treated with normal. *p < .05; **p < .01; ***p < .001, compared with NC group.

According to qRT‐PCR results, circEIF4G2 gene level in DMSO group exhibited no significant difference (*p* > .05, Figure 5), suggesting that DMSO has no effect on the circEIF4G2 gene level in Hela and Siha cells. After allicin treatment, circEIF4G2 gene expression was reduced significantly in allicin‐L, allicin‐M, and allicin‐H groups (*p* < .05, *p* < .01 or *p* < .001, Figure 5). Therefore, allicin‐L, allicin‐M, and allicin‐H decreased the circEIF4G2 gene expression in Hela and Siha cell lines, with best effect noted in allicin‐H.

### 3.6 Effect of circEIF4G2 on CC cell proliferation inhibited by allicin

**FIGURE 6:**
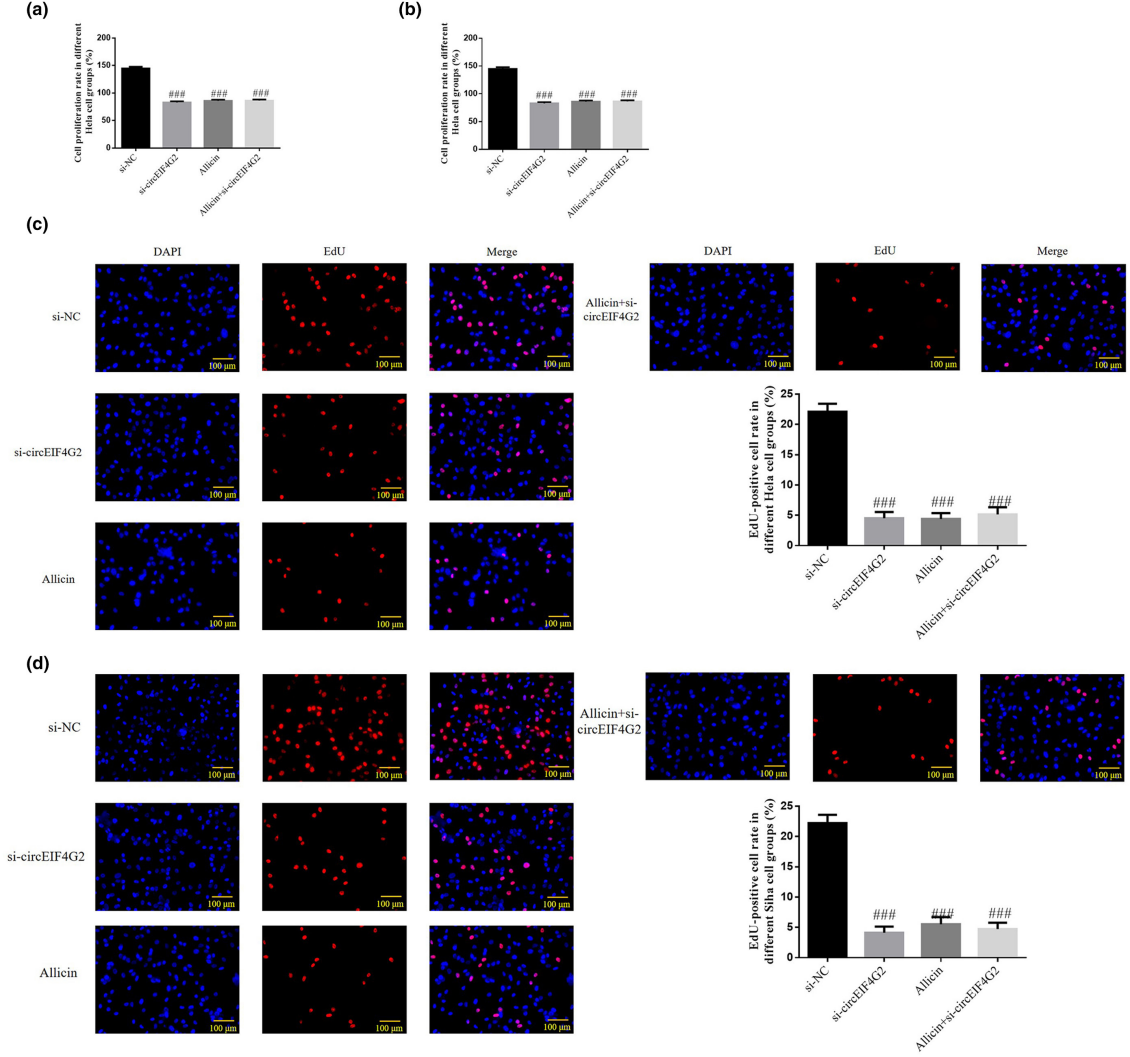
Effect of circEIF4G2 on Allicin inhibiting proliferation of CC cells. (a) Cell proliferation in different Hela cell groups. (b) Cell proliferation in different Siha cell groups. (c) EdU‐positive Hela cell number (×100). (d) EdU‐positive Siha cell number (×100). Allicin, The cells were treated with 80 μ M Allicin; Allicin+si‐circEIF4G2, The cell transfected with si‐circEIF4G2 and treated with 80 μ M Allicin; si‐circEIF4G2, The cells were transfected with si‐circEIF4G2; si‐NC, The cells were transfected with si‐NC (negative control); ^###^ *p* < .001, compared with si‐NC.

According to CCK‐8 assay and EdU staining results, the cell proliferation rate (*p* < .001, Figure 6a,b) and EdU‐positive cell count (*p* < .001, Figure 6c,d) decreased significantly in si‐circEIF4G2, allicin, and allicin+si‐circEIF4G2 groups. However, both showed no significant differences between these three groups (*p* > .05, Figure 6). Therefore, allicin might regulate circEIF4G2 to reduce CC cell proliferation.

### 3.7 Effect of circEIF4G2 CC on cell apoptosis promoted by allicin

**FIGURE 7:**
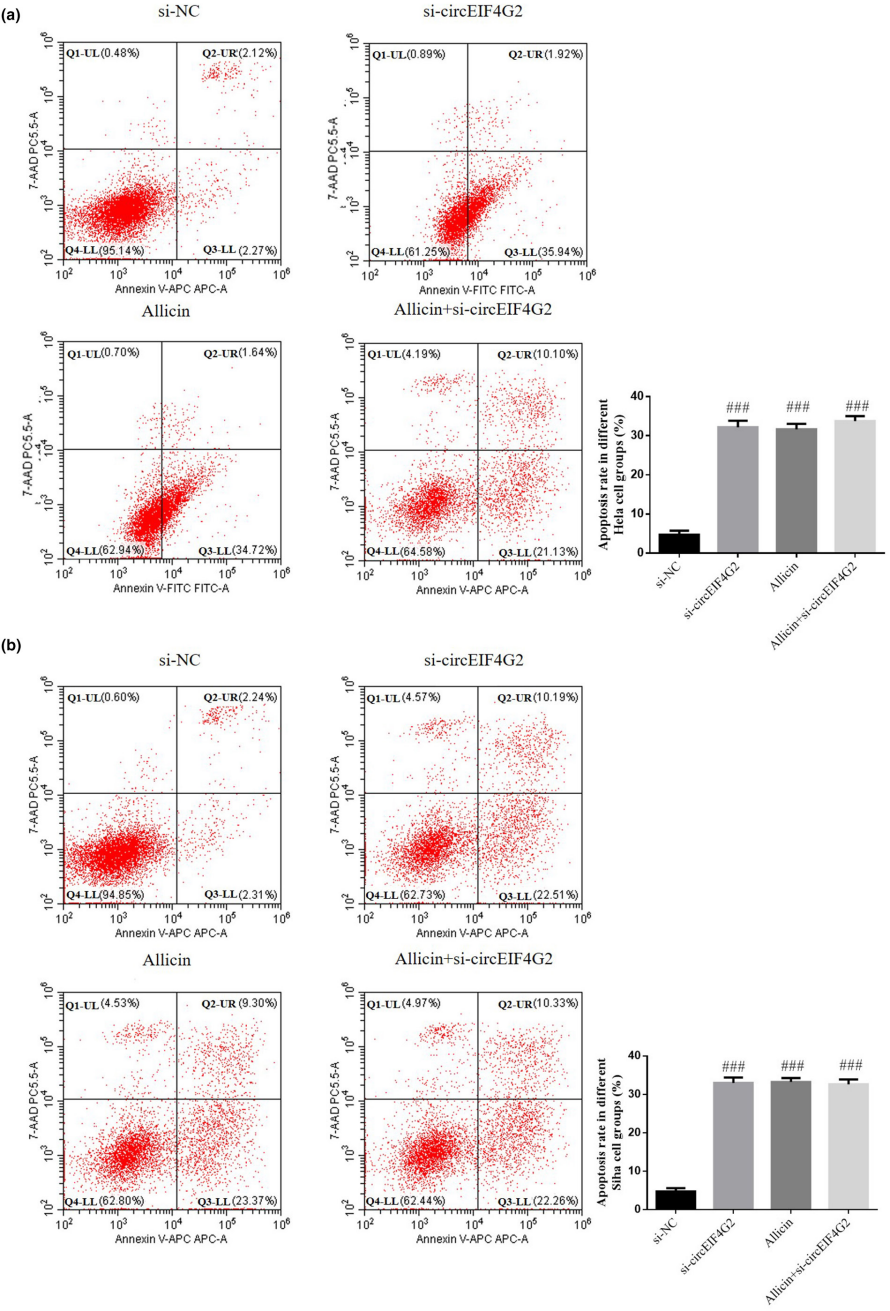
Effect of circEIF4G2 on Allicin promoting apoptosis of CC cells. (a) Cell apoptosis rate in different Hela cell groups. (b) Cell apoptosis rate in different Siha cell groups. Allicin, The cells were treated with 80 μM Allicin; Allicin+si‐circEIF4G2, The cell transfected with si‐circEIF4G2 and treated with 80 μM Allicin; si‐circEIF4G2, The cells were transfected with si‐circEIF4G2; si‐NC, The cells were transfected with si‐NC (negative control); ^###^ *p* < .001, compared with si‐NC.

Flow cytometry revealed apoptotic rate increased significantly in si‐circEIF4G2, allicin, and allicin+si‐circEIF4G2 groups (*p* < .001, respectively, Figure 7a,b). However, it was not significantly different between such groups (*p* > .05, Figure 7). Therefore, allicin might regulate circEIF4G2 to increase CC cell apoptosis.

### 3.8 Role of circEIF4G2 in CC cells' invasive capability inhibited by allicin

**FIGURE 8:**
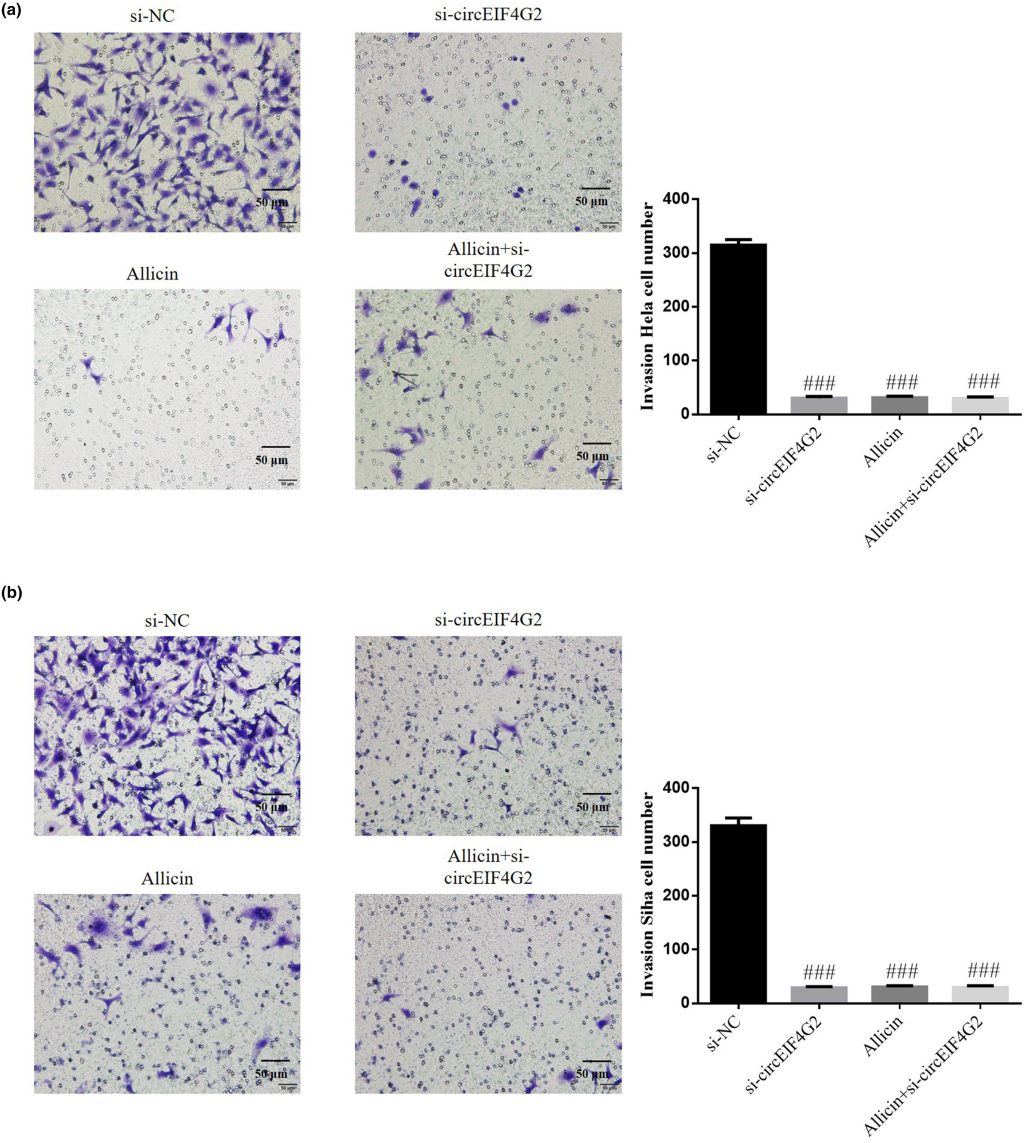
Role of circEIF4G2 in Allicin inhibiting invasive capability of CC cells. (a) Invasion Hela cell number by transwell assay (×100). (b) Invasion Siha cell number by transwell assay (×100). Allicin, The cells were treated with 80 μ M Allicin; Allicin+si‐circEIF4G2, The cell transfected with si‐circEIF4G2 and treated with 80 μ M Allicin; si‐circEIF4G2, The cells were transfected with si‐circEIF4G2; si‐NC, The cells were transfected with si‐NC (negative control). ^###^ *p* < .001, compared with si‐NC.

Transwell assay demonstrated that invasive cell number decreased significantly in si‐circEIF4G2, allicin, and allicin+si‐circEIF4G2 groups (each group: *p* < .001, Figure 8a,b). However, it was not significantly different between these groups (*p* > .05, Figure 8). Thus, allicin might regulate circEIF4G2 to reduce CC cell invasion ability.

### 3.9 Role of circEIF4G2 in CC cell's migration capability inhibited by allicin

**FIGURE 9:**
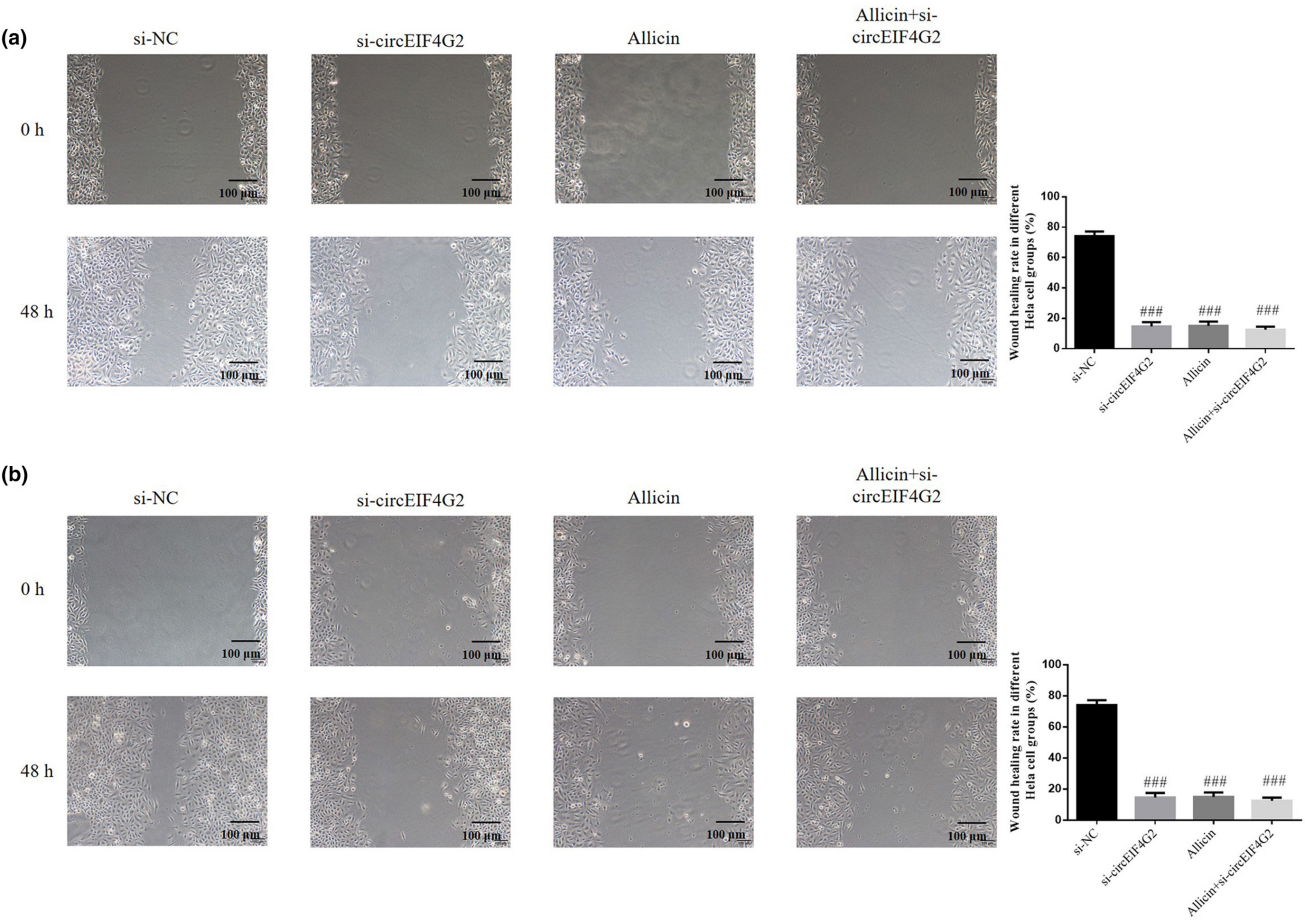
Role of circEIF4G2 in Allicin inhibiting migration capability of CC cells. (a) Wound healing rate in different Hela cell groups (×100). (b) Wound healing rate in different Siha cell groups (×100). Allicin, The cells were treated with 80 μ M Allicin; Allicin+si‐circEIF4G2, The cell transfected with si‐circEIF4G2 and treated with 80 μ M Allicin; si‐circEIF4G2, The cells were transfected with si‐circEIF4G2; si‐NC, The cells were transfected with si‐NC (negative control). ^###^ *p* < .001, compared with si‐NC.

Based on the wound healing assay, wound healing rate decreased significantly in the si‐circEIF4G2, allicin, and allicin+si‐circEIF4G2 groups (each group: *p* < .001, Figure 9a,b). However, it showed no significant differences between such groups (*p* > .05, Figure 9). Therefore, allicin might regulate circEIF4G2 to decrease CC cell migration ability.

### 3.10 Relevant gene levels

**FIGURE 10:**
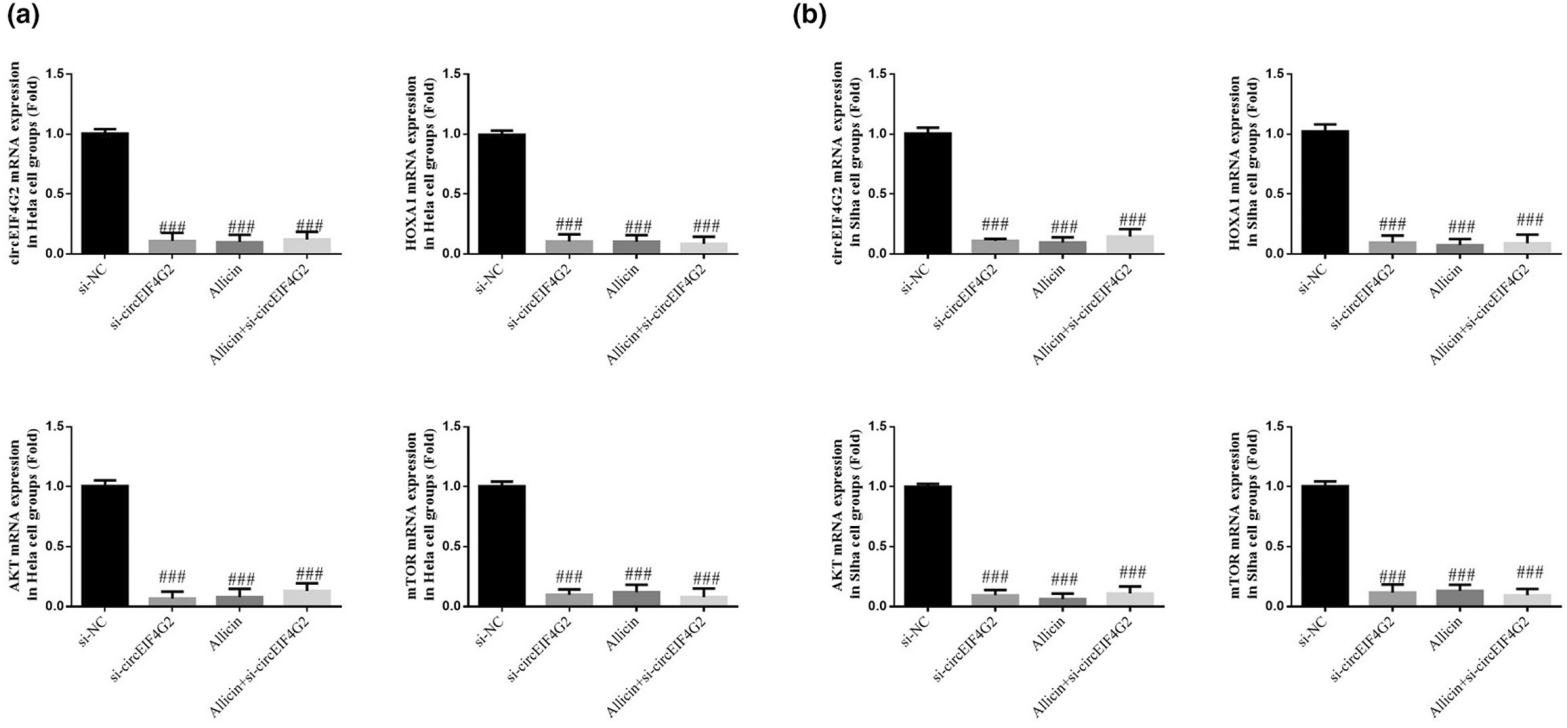
Relative mRNA expression by qRT‐PCR assay. (a) Relative gene expression in different Hela cell groups. (b) Relative gene expression in different Siha cell groups. Allicin, The cells were treated with 80 μ M Allicin; Allicin+si‐circEIF4G2, The cell transfected with si‐circEIF4G2 and treated with 80 μ M Allicin; si‐circEIF4G2, The cells were transfected with si‐circEIF4G2; si‐NC, The cells were transfected with si‐NC (negative control). ^###^ *p* < .001, compared with si‐NC.

According to qRT‐PCR results, levels of circEIF4G2, HOXA1, AKT, and mTOR genes decreased significantly in the si‐circEIF4G2, allicin, and allicin+si‐circEIF4G2 groups (each group: *p* < .001, Figure 10a,b). However, they did not show significant differences between these groups (*p* > .05, Figure 10).

### 3.11 Relevant protein levels

**FIGURE 11:**
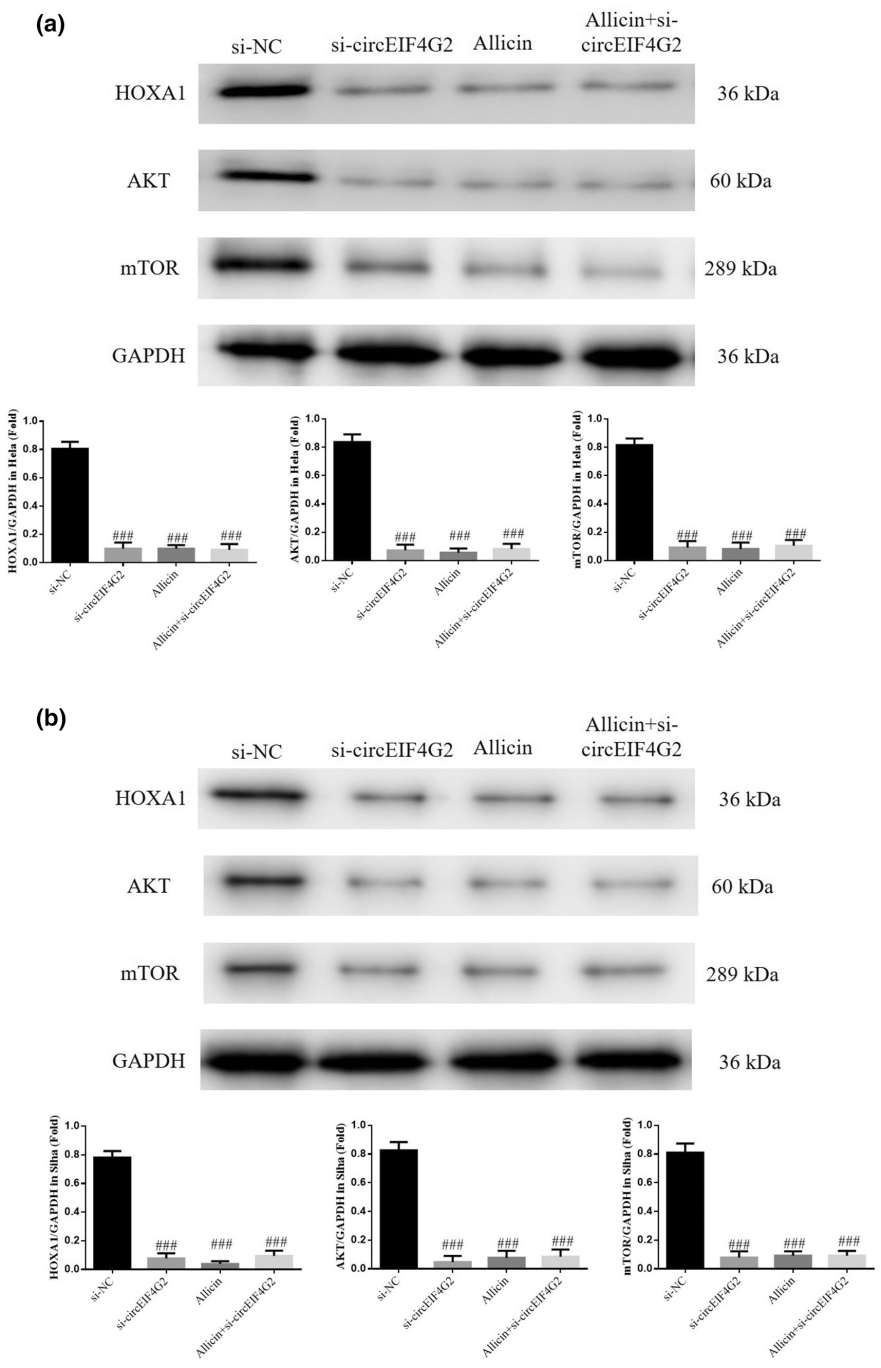
Relative proteins expression by WB assay. (a) Relative protein expression in different Hela cell groups. (b) Relative protein expression in different Siha cell groups. Allicin, The cells were treated with 80 μ M Allicin; Allicin+si‐circEIF4G2, The cell transfected with si‐circEIF4G2 and treated with 80 μ M Allicin; si‐circEIF4G2, The cells were transfected with si‐circEIF4G2; si‐NC, The cells were transfected with si‐NC (negative control). ^###^ *p* < .001, compared with si‐NC.

WB results revealed HOXA1, AKT, and mTOR protein levels decreased significantly in the si‐circEIF4G2, allicin, and allicin+si‐circEIF4G2 groups (each group: *p* < .001, Figure 11a,b). However, their decrease did not significantly differ between these groups (*p* > .05, Figure 11).

## 4 DISCUSSION

This study may be the first to use allicin at different concentrations to suppress Hela and Siha cells and detect proliferation rates in different treatment groups. Results showed allicin treatment significantly inhibited proliferation of Hela and Siha cells, especially at high concentrations. Excessive cell proliferation can promote tumor growth, distant metastasis, and local invasion, which are the main limiting factors in the clinical treatment of CC. Reducing the invasion and metastasis rates of tumor cells can improve clinical efficacy (Liu et al., 2018; Xie et al., 2022). Allicin is an herb extract that was previously reported to have antitumor effects against lung cancer (Bai et al., 2023), but its effects on CC are still unclear. Present study revealed allicin significantly reduced invasion and metastasis rates of Hela and Siha cells, with excellent inhibitory effects at high concentrations. Allicin, which is the main active ingredient of garlic, has various effects, including antitumor, antibacterial, anti–free radical, anti‐inflammatory, and regulatory effects on the body's immune system (Zhou et al., 2022). It also has a certain inhibitory effect on the invasion and metastasis of various tumor cells, such as liver cancer cells (Alamir & Patil, 2021). Allicin has been confirmed to increase the rupture of liver cancer cell nuclei and promote apoptosis (Chu et al., 2013; Zou et al., 2016). In our study, the proliferation, invasion, and migration capabilities of Hela and Siha cells were all significantly inhibited after allicin treatment, while their apoptotic rates increased significantly. In addition, high‐dose allicin presented the best inhibitory effect. Further detection revealed that the expression of the circEIF4G2 gene was inhibited significantly.

Increasing evidence suggests that circRNA disorders are related to the occurrence and development of cancers and other diseases (Dragomir & Calin, 2018; Li et al., 2018). Compared with linear RNAs, circRNAs exhibit higher stability because of their closed‐loop structure and resistance to RNase R (Zhang et al., 2021). Some circRNA levels in various human cells present tissue and disease specificity. They are also increased in brain tissues, especially in synapses. Owing to advances in circRNA microarrays and bioinformatics analysis, many new circRNAs and their functions have been identified. For example, Chen et al. (Fu et al., 2017) conducted a circRNA microarray analysis and found that the hsa_cir_0128298 levels increased in hepatocellular carcinoma (HCC) tissues, suggesting that this circRNA can be used as a new diagnostic and prognostic biomarker for HCC. Wang et al. (Wang & Li, 2018) also proved that circ_0067934 is an oncogene and that its expression correlates with a poor prognosis in patients with non‐small‐cell lung cancer. Furthermore, our previous work (Mao et al., 2019) revealed that circEIF4G2 has a promoting effect on CC. The present study then confirmed that reducing the expression of circEIF4G2 can effectively mitigate CC cells' biological activities. In addition, the transfection of si‐circEIF4G2 into Hela and Siha cells significantly reduced the activities of these cells, but when this transfection was combined with allicin intervention, the anticancer effect was not enhanced. Therefore, allicin may exert its anticancer effect by inhibiting circEIF4G2.

The abnormally high expression of the HOXA1/AKT/mTOR signaling pathway may be one of the key factors leading to tumor occurrence and development (Ma et al., 2022; Zhang et al., 2019). In this study, circEIF4G2 was decreased in Hela and Siha cells, CC cell activities were inhibited, and the HOXA1/AKT/mTOR signaling pathway was significantly inhibited. Based on previous research results, circEIF4G2 has been confirmed to regulate HOXA1 (Mao et al., 2019).

In conclusion, allicin can inhibit CC cells' biological activities by inhibiting circEIF4G2 and down‐regulating downstream HOXA1/AKT/mTOR signaling pathway activities.

## AUTHOR CONTRIBUTIONS

**Mao Yifan:** Formal analysis (equal); funding acquisition (equal); investigation (equal); software (equal); supervision (equal); validation (equal). **Xu Rui:** Funding acquisition (equal); software (equal); supervision (equal); validation (equal). **Li Yuan:** Investigation (equal); validation (equal); writing – review and editing (equal). **Jiang Feiyun:** Formal analysis (equal); funding acquisition (equal); investigation (equal); visualization (equal); writing – original draft (equal).

## FUNDING INFORMATION

Our work was approved by the 2021 Teaching Hospital of Wannan Medical College (No. JXYY202103), the 2021 Wuhu Second People's Hospital Major Project (No. JC2021A04), and the “Hua Tuo Plan” of Wuhu Municipal Health Commission in 2022.

## CONFLICT OF INTEREST STATEMENT

The authors declare that they have no competing interests.

## Data Availability

The results of this study can be provided at the request of the corresponding author, and are not publicly available for privacy or ethical reasons.

## References

[fsn33935-bib-0001] Alamir, A. H. , & Patil, S. (2021). Allicin could potentially alleviate Oral cancer pain by inhibiting "pain mediators" TNF‐alpha, IL‐8, and endothelin. Current Issues in Molecular Biology, 43(1), 187–196. 34071008 10.3390/cimb43010016 PMC8929120

[fsn33935-bib-0002] Bai, X. , Cheng, Y. , Wan, H. , Li, S. , Kang, X. , & Guo, S. (2023). Natural compound allicin containing Thiosulfinate moieties as transmembrane protein 16A (TMEM16A) Ion Channel inhibitor for food adjuvant therapy of lung cancer [J]. Journal of Agricultural and Food Chemistry, 71(1), 535–545. 36574498 10.1021/acs.jafc.2c06723

[fsn33935-bib-0003] Chen, L. , Wang, C. , Sun, H. , Wang, J. , Liang, Y. , Wang, Y. , & Wong, G. (2021). The bioinformatics toolbox for circRNA discovery and analysis. Briefings in Bioinformatics, 22(2), 1706–1728. 32103237 10.1093/bib/bbaa001 PMC7986655

[fsn33935-bib-0004] Chen, R. X. , Liu, H. L. , Yang, L. L. , Kang, F. H. , Xin, L. P. , Huang, L. R. , Guo, Q. F. , & Wang, Y. L. (2019). Circular RNA circRNA_0000285 promotes cervical cancer development by regulating FUS. European Review for Medical and Pharmacological Sciences, 23(20), 8771–8778. 31696463 10.26355/eurrev_201910_19271

[fsn33935-bib-0005] Chu, Y. L. , Ho, C. T. , Chung, J. G. , Raghu, R. , Lo, Y. C. , & Sheen, L. Y. (2013). Allicin induces anti‐human liver cancer cells through the p53 gene modulating apoptosis and autophagy. Journal of Agricultural and Food Chemistry, 61(41), 9839–9848. 24059278 10.1021/jf403241s

[fsn33935-bib-0006] Dragomir, M. , & Calin, G. A. (2018). Circular RNAs in cancer ‐ lessons learned from microRNAs. Frontiers in Oncology, 8, 179. 29911069 10.3389/fonc.2018.00179 PMC5992376

[fsn33935-bib-0007] Fu, L. , Yao, T. , Chen, Q. , Mo, X. , Hu, Y. , & Guo, J. (2017). Screening differential circular RNA expression profiles reveals hsa_circ_0004018 is associated with hepatocellular carcinoma. Oncotarget, 8(35), 58405–58416. 28938566 10.18632/oncotarget.16881 PMC5601662

[fsn33935-bib-0008] Igissinov, N. , Igissinova, G. , Telmanova, Z. , Bilyalova, Z. , Kulmirzayeva, D. , Kozhakhmetova, Z. , Urazova, S. , Turebayev, D. , Nurtazinova, G. , Omarbekov, A. , Almabayeva, A. , Bukeyeva, Z. , Tarzhanova, D. , Moldabayeva, A. , Znanaliyeva, M. , Kazbekova, A. , Openko, V. , Kozhakhmetov, S. , & Kuandykov, Y. (2021). New trends of cervical cancer incidence in Kazakhstan. Asian Pacific Journal of Cancer Prevention, 22(4), 1295–1304. 33906325 10.31557/APJCP.2021.22.4.1295 PMC8325133

[fsn33935-bib-0009] Li, M. , Ding, W. , Sun, T. , Tariq, M. A. , Xu, T. , Li, P. , & Wang, J. (2018). Biogenesis of circular RNAs and their roles in cardiovascular development and pathology. The FEBS Journal, 285(2), 220–232. 28783257 10.1111/febs.14191

[fsn33935-bib-0010] Liu, Y. , Yang, Y. , Li, L. , Liu, Y. , Geng, P. , Li, G. , & Song, H. (2018). LncRNA SNHG1 enhances cell proliferation, migration, and invasion in cervical cancer. Biochemistry and Cell Biology, 96(1), 38–43. 28930646 10.1139/bcb-2017-0188

[fsn33935-bib-0011] Ma, L. , Zhang, M. , Cao, F. , Han, J. , Han, P. , Wu, Y. , Deng, R. , Zhang, G. , An, X. , Zhang, L. , Song, Y. , & Cao, B. (2022). Effect of MiR‐100‐5p on proliferation and apoptosis of goat endometrial stromal cell in vitro and embryo implantation in vivo. Journal of Cellular and Molecular Medicine, 26(9), 2543–2556. 35411593 10.1111/jcmm.17226 PMC9077292

[fsn33935-bib-0012] Maitisha, G. , Aimaiti, M. , An, Z. , & Li, X. (2021). Allicin induces cell cycle arrest and apoptosis of breast cancer cells in vitro via modulating the p53 pathway. Molecular Biology Reports, 48(11), 7261–7272. 34626309 10.1007/s11033-021-06722-1

[fsn33935-bib-0013] Mao, Y. , Zhang, L. , & Li, Y. (2019). circEIF4G2 modulates the malignant features of cervical cancer via the miR‐218/HOXA1 pathway. Molecular Medicine Reports, 19(5), 3714–3722. 30896864 10.3892/mmr.2019.10032 PMC6471440

[fsn33935-bib-0014] Shaswati, M. , Oeishy, F. H. , Mumu, S. B. , Zahid, M. Z. I. , Hossain, M. , Haque, M. A. , Reza, H. M. , & Mostaid, M. S. (2023). Polymorphisms of the interleukin‐6 (IL‐6) gene contribute to cervical cancer susceptibility in Bangladeshi women: A case‐control study. Health Science Reports, 6(5), e1238. 37152226 10.1002/hsr2.1238 PMC10155201

[fsn33935-bib-0015] Su, Y. , Lv, X. , Yin, W. , Zhou, L. , Hu, Y. , Zhou, A. , & Qi, F. Z. (2019). CircRNA Cdr1as functions as a competitive endogenous RNA to promote hepatocellular carcinoma progression. Aging (Albany NY), 11(19), 8183–8203. 31581132 10.18632/aging.102312 PMC6814590

[fsn33935-bib-0016] Tomizawa, K. , Kaminuma, T. , Murata, K. , Noda, S. E. , Irie, D. , Kumazawa, T. , Oike, T. , & Ohno, T. (2020). FIGO 2018 staging for cervical cancer: Influence on stage distribution and outcomes in the 3D‐image‐guided brachytherapy era. Cancers (Basel), 12(7), 1770. 32630799 10.3390/cancers12071770 PMC7408064

[fsn33935-bib-0017] Wang, J. , & Li, H. (2018). CircRNA circ_0067934 silencing inhibits the proliferation, migration and invasion of NSCLC cells and correlates with unfavorable prognosis in NSCLC. European Review for Medical and Pharmacological Sciences, 22(10), 3053–3060. 29863250 10.26355/eurrev_201805_15063

[fsn33935-bib-0018] Xie, Q. , Li, Z. , Luo, X. , Wang, D. , Zhou, Y. , Zhao, J. , Gao, S. , Yang, Y. , Fu, W. , Kong, L. , & Sun, T. (2022). piRNA‐14633 promotes cervical cancer cell malignancy in a METTL14‐dependent m6A RNA methylation manner. Journal of Translational Medicine, 20(1), 51. 35093098 10.1186/s12967-022-03257-2 PMC8802215

[fsn33935-bib-0019] Yu, L. , & Liu, Y. (2019). circRNA_0016624 could sponge miR‐98 to regulate BMP2 expression in postmenopausal osteoporosis. Biochemical and Biophysical Research Communications, 516(2), 546–550. 31235259 10.1016/j.bbrc.2019.06.087

[fsn33935-bib-0020] Zhang, L. , Liu, X. L. , Yuan, Z. , Cui, J. , & Zhang, H. (2019). MiR‐99a suppressed cell proliferation and invasion by directly targeting HOXA1 through regulation of the AKT/mTOR signaling pathway and EMT in ovarian cancer. European Review for Medical and Pharmacological Sciences, 23(11), 4663–4672. 31210292 10.26355/eurrev_201906_18046

[fsn33935-bib-0021] Zhang, P. , & Dai, M. (2022). CircRNA: A rising star in plant biology. Journal of Genetics and Genomics, 49(12), 1081–1092. 35644325 10.1016/j.jgg.2022.05.004

[fsn33935-bib-0022] Zhang, S. , Sun, J. , Gu, M. , Wang, G. , & Wang, X. (2021). Circular RNA: A promising new star for the diagnosis and treatment of colorectal cancer. Cancer Medicine, 10(24), 8725–8740. 34796685 10.1002/cam4.4398 PMC8683543

[fsn33935-bib-0023] Zhou, Y. , Li, X. , Luo, W. , Zhu, J. , Zhao, J. , Wang, M. , Sang, L. , Chang, B. , & Wang, B. (2022). Allicin in digestive system cancer: From biological effects to clinical treatment. Frontiers in Pharmacology, 13, 903259. 35770084 10.3389/fphar.2022.903259 PMC9234177

[fsn33935-bib-0024] Zou, X. , Liang, J. , Sun, J. , Hu, X. , Lei, L. , Wu, D. , & Liu, L. (2016). Allicin sensitizes hepatocellular cancer cells to anti‐tumor activity of 5‐fluorouracil through ROS‐mediated mitochondrial pathway. Journal of Pharmacological Sciences, 131(4), 233–240. 27177453 10.1016/j.jphs.2016.04.017

